# Comparison of the Alvarado Score with Alternative Diagnostic Tools in Pediatric Acute Appendicitis: A Literature Review

**DOI:** 10.3390/pediatric18040115

**Published:** 2026-08-18

**Authors:** Paul Tchouala Tchakoute, Alin Iuhas, Vlad-Ionuț Nechita, Andrei Vasile Pașcalău, Buzdugan Mihaela Ștefania, Ion Cosmin Puia

**Affiliations:** 1 Doctoral School of Biomedical Sciences, University of Oradea, 410073 Oradea, Romania; 2 Department of Pediatric Surgery and Orthopedics, Bihor County Emergency Clinical Hospital, 410469 Oradea, Romania; 3 Department of Medical Disciplines, Faculty of Medicine and Pharmacy, University of Oradea, 410087 Oradea, Romania; 4 Department of Fundamental Sciences, Discipline of Medical Informatics, Research Methodology and Data Analysis, Faculty of Nursing and Health Sciences (FAMSS), “Iuliu Hațieganu” University of Medicine and Pharmacy, 400012 Cluj-Napoca, Romania; 5 Department of Morphological Disciplines, Faculty of Medicine and Pharmacy, University of Oradea, 410087 Oradea, Romania; 6 Department of Pathology, Bihor County Emergency Clinical Hospital, 410469 Oradea, Romania; 7 Department of Premedical Disciplines, Faculty of Medicine and Pharmacy, University of Oradea, 410087 Oradea, Romania; 8 Department of Surgery, “Octavian Fodor” Regional Institute of Gastroenterology and Hepatology, 400162 Cluj-Napoca, Romania; 9 Department of Surgery, “Iuliu Hațieganu” University of Medicine and Pharmacy, 400162 Cluj-Napoca, Romania

**Keywords:** acute appendicitis, pediatrics, Alvarado score, AIR score, PAS, pARC, diagnostic accuracy

## Abstract

Background: Acute appendicitis is a leading pediatric surgical emergency. Timely diagnosis remains challenging, particularly in young children with atypical presentations. Clinical evaluation alone yields variable diagnostic performance, prompting the use of risk stratification tools. Objective: To evaluate and compare the Alvarado score against alternative clinical scoring systems, laboratory biomarkers, and imaging modalities in pediatric acute appendicitis. Methods: A narrative literature review with structured selection elements was conducted across PubMed, Scopus, and Web of Science for studies published between 2016 and 2026. Results: The Alvarado score demonstrates moderate diagnostic accuracy in children, with good sensitivity but limited specificity (57–76%) at standard cut-offs (≥7). Pediatric-specific scores like the Pediatric Appendicitis Score (PAS) offer higher sensitivity, while the Appendicitis Inflammatory Response (AIR) score provides superior specificity and better identifies complicated appendicitis (including gangrene, phlegmon, and perforation). Continuous risk models, notably the Pediatric Appendicitis Risk Calculator (pARC), outperform fixed additive scores in pediatric cohorts. Combining clinical scores with C-reactive protein, neutrophil-to-lymphocyte ratio, and selective ultrasonography enhances diagnostic precision. Computed tomography reserved for equivocal cases further optimizes diagnostic yield. Conclusion: The Alvarado score should not be used as a standalone tool. A structured, stepwise multimodal approach integrating clinical scores (such as pARC or PAS), inflammatory biomarkers, and selective imaging optimizes diagnostic accuracy and supports timely surgical decision-making in children.

## 1. Introduction

Acute appendicitis represents one of the most common pediatric surgical emergencies worldwide and remains a leading cause of acute abdominal pain requiring urgent surgical intervention in children. Recent studies have highlighted the ongoing diagnostic challenges and variability in the performance of clinical scoring systems across different patient populations [1,2]. Despite advances in diagnostic strategies, accurate and timely diagnosis continues to be challenging, particularly in younger patients, due to atypical clinical presentations and rapid disease progression [3,4,5].

The lifetime risk of appendicitis is estimated at approximately 7–8% in the general population, with a significant proportion of cases occurring during childhood and adolescence [6,7]. In pediatric populations, especially in children under five years of age, diagnosis is particularly difficult due to nonspecific symptoms, limited communication abilities, and overlap with other common pediatric conditions [8,9]. Consequently, delayed diagnosis remains common and is associated with an increased risk of complications such as perforation, reported in up to 30–50% of cases in younger children [4,10].

Traditionally, the diagnosis of acute appendicitis has relied on clinical evaluation, including patient history and physical examination. Clinical assessment alone carries inherent diagnostic uncertainty. These limitations have led to the development of clinical scoring systems designed to improve diagnostic performance and standardize patient evaluation. Historical and unstratified surgical series reported negative appendectomy rates of 15% to 30% [8,11]; however contemporary practice integrating risk stratification scores and protocolized, selective imaging has substantially reduced these rates in modern pediatric centers.

The Alvarado score, first described in 1986, continues to represent one of the most widely used diagnostic tools in both adult and pediatric populations [12]. It combines clinical symptoms, physical examination findings, and laboratory parameters into a simple scoring system. Although widely used, several studies and meta-analyses have shown that the Alvarado score has moderate diagnostic accuracy, particularly in pediatric populations [10,11,13,14]. As a result, reliance on the Alvarado score alone may contribute to both to minimize negative appendectomy rates (defined by histological confirmation of a normal appendix) and reduce diagnostic delays. To address these limitations, several alternative scoring systems have been developed, including the Pediatric Appendicitis Score (PAS), Appendicitis Inflammatory Response (AIR) score, Raja-Isteri-Pengiran-Anak-Saleha (RIPASA) score, and Tzanakis score [15,16,17,18]. The PAS, initially described by Samuel [15], has demonstrated high sensitivity in multiple pediatric studies [19,20]. In contrast, the AIR score appears to exhibit improved specificity and enhanced ability to differentiate between complicated (encompassing gangrenous, suppurative, and perforated disease) and uncomplicated appendicitis, largely due to the inclusion of inflammatory biomarkers such as C-reactive protein, although results may vary depending on the study population [21,22,23,24]. Similarly, the RIPASA and Tzanakis scores incorporate additional clinical and imaging parameters and have demonstrated improved or comparable diagnostic performance in selected patient populations [1,25,26].

Beyond clinical scoring systems, laboratory biomarkers play a significant role in the diagnostic process. Commonly used parameters include white blood cell count (WBC), C-reactive protein (CRP), and the neutrophil-to-lymphocyte ratio (NLR), all of which reflect the inflammatory response. Although none of these markers alone is sufficiently specific, their combination with clinical scores improves diagnostic accuracy and risk stratification [11,27,28].

Alongside laboratory findings, imaging modalities play an important role in the diagnostic evaluation of suspected appendicitis in children. Ultrasonography is widely used as the first-line imaging technique due to its safety, availability, and absence of ionizing radiation [29,30]. However, its clinical performance is operator-dependent and may be limited in certain scenarios. Computed tomography (CT) provides higher diagnostic precision and is particularly useful in equivocal cases, but its use in pediatric patients is limited by concerns regarding radiation exposure [31].

Given the wide range of available diagnostic tools, there is ongoing debate regarding the optimal approach for diagnosing pediatric acute appendicitis. While numerous studies have evaluated individual scoring systems or diagnostic modalities, fewer have provided comprehensive comparisons that integrate clinical scores, laboratory biomarkers, and imaging techniques. Therefore, the aim of this narrative review is to evaluate and compare the diagnostic performance of the Alvarado score with alternative clinical scoring systems, laboratory biomarkers, and imaging modalities in pediatric acute appendicitis, highlighting their strengths, limitations, and role within a multimodal diagnostic framework.

## 2. Materials and Methods

This study is a narrative review with elements of structured literature selection. For the purpose of this review, the pediatric population was strictly defined as patients under 18 years of age (<18 years). Relevant studies were identified through database searches using PubMed, Scopus, and Web of Science, focusing on pediatric acute appendicitis and clinical diagnostic scoring tools. Studies published in English between 2016 and 2026 were primarily considered. Database searches utilized combinations of Boolean operators and key terms, including: (“Alvarado score” OR “Pediatric Appendicitis Score” OR “PAS” OR “AIR score” OR “pARC”) AND (“pediatric appendicitis” OR “children”) AND (“ultrasound” OR “computed tomography” OR “biomarkers”). A narrative synthesis design with structured selection elements was adopted rather than a formal PRISMA systematic review because the primary objective was to provide a pragmatic, clinically oriented diagnostic algorithm and critical discussion for practitioners rather than a restricted statistical meta-analysis. Adult-only cohorts were excluded from the primary evidence synthesis and utilized solely to provide historical derivation background for specific scoring systems. Additionally, seminal and widely cited studies were included to provide background context and support key concepts. A structured screening process was applied, including evaluation of titles and abstracts followed by full-text assessment to identify the most relevant articles. Studies with insufficient relevance or methodological limitations were excluded, resulting in the inclusion of a subset of key studies in the core analysis. Complementary references were incorporated to support specific concepts, provide methodological context, and enhance the comprehensiveness of the review. This approach ensured the inclusion of relevant and recent evidence, thereby contributing to the consistency and reliability of the findings.

## 3. Results

### 3.1. Clinical Findings in Pediatric Appendicitis

Clinical examination remains essential in the assessment of pediatric appendicitis, although its diagnostic value varies depending on patient age and clinical context. Several clinical features, including right lower quadrant tenderness, signs of peritoneal irritation, and neutrophilia (>75%), have been identified as significant predictors of appendicitis requiring surgical intervention, with reported odds ratios ranging from 12.57 to 21.07 across studies [3].

More structured approaches, including clinical scoring systems such as the Alvarado score and PAS, provide improved diagnostic consistency compared to clinical evaluation alone [5]. The reported diagnostic performance of clinical scoring systems varies considerably depending on the selected cut-off threshold, study design, and age distribution. When evaluated at standard published thresholds in pediatric validation cohorts, the Alvarado score demonstrates moderate diagnostic accuracy, with reported sensitivities ranging from 72% to 88% and specificities from 57% to 76% at the conventional cut-off of ≥7 (Table 1). In comparison, the Pediatric Appendicitis Score evaluated at a cut-off of ≥7 shows higher sensitivity (80–95%) but moderate specificity (60–70%). The Appendicitis Inflammatory Response score (cut-off ≥ 5 for moderate/high risk) provides a more balanced profile with superior specificity (78–90%). The RIPASA score (cut-off ≥ 7.5) and Tzanakis score (cut-off ≥ 8) also report high sensitivities and specificities; however, their primary evidence base stems largely from adult or mixed populations, requiring cautious interpretation when applied to children [10,13,14,21,22,23].

It is important to note that several widely evaluated clinical scores were originally developed and derived in adult or general surgical populations. The AIR score was derived in a general surgical cohort, while the RIPASA and Tzanakis scores were initially evaluated in adult-dominated populations (e.g., mean age ~28 years in the original Tzanakis cohort). In this review, evidence derived from adult cohorts is treated strictly as historical context, whereas our primary comparative synthesis focuses exclusively on studies that validated these scores within dedicated pediatric cohorts (<18 years).

The data highlight the variability in sensitivity (72–96%) and specificity (57–90%) across different scoring systems. These findings show the heterogeneity in diagnostic performance and underscore the limitations of relying on a single clinical tool.

Rebound tenderness and fever are associated with higher specificity for appendicitis, while symptoms such as anorexia, nausea, and RLQ tenderness are associated with higher sensitivity. Reported specificity values for signs of peritoneal irritation range up to approximately 80–90% in several studies, whereas sensitivity remains more variable depending on the clinical presentation.

Additional clinical signs, including local guarding and the Rovsing sign, further contribute to diagnostic assessment. In the absence of radiological and laboratory investigations, the Alvarado score may represent a useful clinical tool for the initial assessment of suspected appendicitis [32], although its diagnostic performance remains dependent on the clinical context.

A comprehensive comparison of the components, scoring criteria, total scores, and diagnostic thresholds of commonly used appendicitis scoring systems is presented in Table 2.

This comparison further highlights the variability in included parameters and scoring weights, which may explain the observed differences in diagnostic reliability across scoring systems. These observations may also support the use of a multimodal approach [21,22,23].

Since its introduction, the Alvarado score has been extensively evaluated as a diagnostic tool for appendicitis; however, further studies are required to optimize its diagnostic performance [32]. Although it remains useful in clinical practice, it does not provide definitive criteria for determining the need for surgical intervention [33]. Several studies, including meta-analyses, have indicated that the diagnostic accuracy of the Alvarado score in pediatric patients is moderate, and therefore it should not be used as a standalone diagnostic method [10,13,14].

In patients with intermediate Alvarado scores, the immature granulocyte count has been identified as a potential predictive factor for pediatric appendicitis and may support the selection of patients who could benefit from CT evaluation [34]. In older children with uncomplicated appendicitis, an Alvarado score of seven or higher, particularly when associated with the presence of an appendicolith, has been reported to be linked to a lower likelihood of successful non-operative management [35]. Furthermore, higher Alvarado scores have been found to be associated with an increased risk of postoperative complications following appendectomy [36].

PAS demonstrates higher sensitivity compared to the Alvarado score, whereas the Alvarado score tends to show higher specificity relative to other scoring systems. Additionally, the diagnostic performance of these scoring systems may vary depending on age and sex, with PAS appearing to perform better in adolescent patients [19].

Although the Alvarado and PAS scores are useful in clinical practice, neither is sufficient to definitively diagnose acute appendicitis without adjunct investigations, such as laboratory markers (e.g., CRP) and imaging studies (ultrasonography) [37]. In cases where the appendix is not visualized on ultrasound, the presence of periappendiceal fat echogenicity, in combination with an Alvarado score above six, may further support the diagnosis of appendicitis [29]. Evaluating appendiceal diameter on ultrasonography requires careful clinical context, as a rigid binary threshold of ≥6 mm demonstrates limited specificity and may lead to false-positive diagnoses. An outer appendiceal diameter exceeding 6 mm can occur in non-inflammatory conditions, such as reactive lymphoid hyperplasia or luminal distension by fecal material. Consequently, a three-category diameter model is increasingly recommended in pediatric emergency care: an appendix measuring <6 mm without secondary signs is considered normal; a diameter of 6 to 8 mm represents an equivocal or borderline category requiring correlation with inflammatory markers and clinical evolution; and an outer diameter > 8 mm (or ≥6 mm accompanied by definite secondary signs such as wall hyperemia, peri-appendiceal fat echogenicity, or fluid collections) strongly supports acute appendicitis [30,38].

In several studies, the diagnostic performance of commonly used tools provided considerable variability. The Alvarado score has been linked to a specificity of 36.8% and sensitivity of 89.5%, while WBC has shown a specificity of 51.9% and sensitivity of 86.4%. CRP has demonstrated higher specificity (98.8%) but lower sensitivity (63.2%) in comparison [11]. Furthermore, WBC and CRP values have been significantly associated with the differentiation between complicated (defined by operative/histopathological presence of perforation, abscess, or gangrene) and uncomplicated appendicitis (*p* < 0.001) [11]. Although the Alvarado score remains useful compared to individual laboratory markers, clinical evaluation continues to represent the cornerstone of diagnosis and management [11,39,40,41].

The modified Alvarado score (MAS) has been proposed as a useful tool in emergency settings, particularly when combined with imaging techniques that avoid ionizing radiation, potentially reducing unnecessary referrals and diagnostic delays [39]. The diagnostic performance of MAS has been reported with a sensitivity of 89.4% and specificity of 33.3%, while ultrasonography has been shown to provide higher sensitivity but lower specificity in several studies [40]. The combined use of these approaches may contribute to a reduction in the rate of negative appendectomies.

Among the individual components of the modified Alvarado score, clinical tenderness remains one of the most important diagnostic signs in pediatric appendicitis [41]. Beyond clinical scoring systems, laboratory markers and imaging modalities play a crucial complementary role in the diagnostic process.

### 3.2. Laboratory and Imaging Investigations

In the diagnosis of pediatric acute appendicitis, individual parameters such as the Alvarado score, CRP, or appendiceal diameter should not be used independently. When combined, however, these parameters have been linked to improved diagnostic accuracy and a reduction in the rate of negative appendectomies in several studies [11,27,42]. Furthermore, the combination of the Alvarado score and CRP levels has been reported to enhance diagnostic performance, particularly in patients presenting with abdominal pain [27]. These findings support the use of an integrated diagnostic approach.

The combined diagnostic roles of laboratory and imaging tools are summarized in Table 3.

Computed tomography exhibits high diagnostic validity, but its routine use in children should be limited due to ionizing radiation exposure. Diagnostic pathways should minimize avoidable delays while preserving appropriate confirmation of the diagnosis. Therefore, CT is best utilized selectively as a second-line tool in equivocal or intermediate-risk cases where ultrasonography is non-diagnostic or discordant with clinical parameters [43].

In patients with uncertain clinical scores, CT imaging may be considered to clarify the diagnosis, whereas in low-risk patients, ultrasonography is generally preferred to exclude alternative diagnoses. In most studies, CT demonstrates sensitivity values exceeding 90%, while ultrasonography shows more variable sensitivity, typically ranging between 70% and 90%, depending on operator experience. In high-risk cases, surgical management is typically recommended without delay based on clinical assessment [31].

The AIR score, which incorporates CRP, has been connected to higher specificity compared to the Alvarado score and may contribute to a reduction in the rate of negative appendectomies in several studies [44]. Although neither AIR nor the Alvarado score alone is sufficient for a definitive diagnosis, AIR score appears to be particularly useful in ruling out appendicitis and in differentiating between complicated and uncomplicated cases [23,26].

The Pediatric Appendicitis Risk Calculator (pARC) represents a dedicated pediatric clinical prediction model designed specifically to address the limitations of traditional summed scoring systems in children. Unlike categorical scores that assign fixed integer points to clinical parameters, pARC incorporates age, sex, symptom duration, and specific physical and laboratory findings (including right lower quadrant pain, guarding, rebound tenderness, absolute neutrophil count, and white blood cell count) to generate a continuous, individualized probability of acute appendicitis. Originally developed and validated across large, diverse pediatric cohorts in emergency department settings, pARC has consistently demonstrated superior discrimination and calibration compared to the Alvarado score and PAS. By providing a quantified risk percentage rather than a rigid risk group, pARC enables tailored clinical decision-making, optimizing the selective use of diagnostic imaging such as ultrasonography and computed tomography in pediatric emergency care [21].

PAS has been correlated with improved diagnostic performance in several studies compared to the Alvarado score, particularly in pediatric patients presenting with typical clinical features [20].

NLR has been suggested to be more reliable than individual leukocyte counts in several studies. Elevated NLR, AIR, and MAS have been significantly associated with histopathologically confirmed appendicitis (*p* < 0.01). Moreover, combining NLR with the AIR score has been linked to improved diagnostic performance compared to other combinations, although results may vary across studies [28]. The AIR score is further characterized by higher sensitivity and specificity, along with a lower rate of false-positive results compared with the Alvarado score in several studies [24].

Diagnosis remains particularly challenging, especially in younger children. Clinical scoring systems such as the Alvarado, PAS, and AIR scores may assist in diagnosis, with PAS being associated with improved diagnostic performance when combined with ultrasonography findings [9].

RIPASA score has been identified as a useful diagnostic tool in pediatric appendicitis, particularly in patients presenting with right lower quadrant tenderness. It has been connected to higher sensitivity and overall diagnostic value compared to the Alvarado score in several studies, potentially reducing unnecessary admissions and imaging investigations [25,26,45]. Some studies have also suggested that the RIPASA score may provide improved diagnostic performance compared to the Alvarado and AIR scores, although results vary across different study populations [26].

The Tzanakis score, which incorporates ultrasonography findings, has been associated with high diagnostic accuracy and sensitivity. However, the Alvarado score remains more specific in ruling out appendicitis in several studies. Additionally, the Lintula score has been related to a lower rate of negative appendectomies, although the Tzanakis score appears to provide higher overall diagnostic accuracy in certain clinical settings [1,46].

## 4. Discussion

The diagnosis of acute appendicitis in pediatric patients continues to represent a complex clinical challenge, primarily due to variability in clinical presentation and the limitations of individual diagnostic tools. The current literature indicates that no single scoring system provides sufficient diagnostic accuracy when used independently, emphasizing the importance of a multimodal diagnostic approach. These observations are consistent with the original description of the Alvarado score [1,12] and subsequent meta-analyses reporting its moderate diagnostic performance [13,14].

This interpretation is further supported by the data presented in Table 1, which highlight differences in sensitivity and specificity among scoring systems. In particular, the AIR score is associated with higher specificity, whereas PAS tends to demonstrate higher sensitivity in several studies.

The Alvarado score continues to be widely used in clinical practice due to its simplicity, reproducibility, and accessibility. However, multiple studies included in this review indicate that its diagnostic performance in pediatric populations is moderate. Although it is characterized by relatively high sensitivity, its limited specificity may contribute to unnecessary surgical interventions and increased negative appendectomy rates [10,11,13,27]. This is consistent with the data presented in Table 1, which highlight the role of the Alvarado score as a useful screening tool but also underscore its limited specificity when compared with alternative scoring systems.

Comparative analyses suggest that alternative scoring systems may provide improved diagnostic performance in specific clinical contexts. The AIR score is characterized by higher specificity and improved discrimination between complicated and uncomplicated appendicitis in several studies, although its clinical performance may vary depending on the study population and clinical setting [16,21,22,23,28]. This may be explained, at least in part, by the inclusion of objective inflammatory markers such as C-reactive protein.

Similarly, the PAS, first introduced by Samuel [15], has been reported to be associated with high sensitivity in multiple pediatric studies and may be particularly useful as a screening tool in children and adolescents [19,20]. However, its relatively lower specificity limits its use as a standalone diagnostic method.

In comparison to traditional additive scoring systems, the Pediatric Appendicitis Risk Calculator provides a major methodological advantage by accounting for continuous variables and clinical interactions specific to pediatric populations. Traditional scores like Alvarado and AIR were largely derived from adult or mixed cohorts, whereas pARC was specifically engineered and prospectively validated in pediatric emergency department populations. Evidence shows that pARC outperforms both the Alvarado score and PAS in accurate risk stratification, significantly reducing unnecessary diagnostic imaging while maintaining high sensitivity for appendicitis. Integrating pARC into routine pediatric pathways allows clinicians to replace arbitrary score cut-offs with individualized percentage risks, directly supporting standardized, evidence-based management algorithms.

The RIPASA and Tzanakis scores have demonstrated high diagnostic accuracy in selected adult and mixed cohorts; however, their direct applicability to pediatric appendicitis remains less extensively validated compared to pediatric-specific tools like PAS or pARC. Because scoring systems like AIR, RIPASA, and Tzanakis incorporate adult-derived thresholds and weights, clinicians must exercise caution when applying them to young children. Future prospective pediatric trials are required to establish age-adjusted thresholds before these adult-derived scores can be broadly recommended in pediatric emergency pathways [1,17,18,25,26].

Beyond clinical scoring systems, laboratory parameters play an important complementary role in refining diagnostic accuracy. Parameters such as white blood cell count, C-reactive protein, and the neutrophil-to-lymphocyte ratio reflect the inflammatory response and have been linked to improved diagnostic precision when integrated with clinical scoring systems [11,27,28]. In particular, C-reactive protein has been associated with disease severity and may further contribute to diagnostic stratification when interpreted in relation to symptom duration [11,27,28]. However, none of these biomarkers provides sufficient specificity to serve as a standalone diagnostic test, and their value is maximized when incorporated into a broader, multimodal diagnostic framework.

In addition to laboratory markers, imaging modalities further contribute to improving diagnostic precision and remain a cornerstone in the evaluation of suspected appendicitis in pediatric patients. Ultrasonography is universally recommended as the initial imaging modality due to its safety and absence of radiation [11,27,29,38,47]. However, when sonographic findings are non-diagnostic, institutional algorithms increasingly endorse rapid, non-contrast MRI as the preferred secondary cross-sectional tool to avoid ionizing radiation while maintaining diagnostic accuracy comparable to CT. Computed tomography should be reserved for centers without immediate MRI capabilities or when MRI is clinically unfeasible. In clinical practice, a stepwise diagnostic approach, incorporating clinical scoring, laboratory evaluation, first-line ultrasonography, and selective non-ionizing cross-sectional imaging (MRI) safeguards against unnecessary radiation exposure while minimizing negative appendectomy rates. Computed tomography provides higher sensitivity and specificity and is particularly useful when clinical and laboratory findings are discordant or when ultrasonography results are inconclusive [38,44]. These observations are consistent with previous studies evaluating clinical assessment, imaging strategies, and diagnostic pathways in pediatric appendicitis [47,48,49,50,51,52]. Regarding imaging criteria, sole reliance on a binary appendiceal cut-off of 6 mm on ultrasonography should be avoided in children. Adopting a three-category model—where appendiceal measurements between 6 and 8 mm are interpreted as equivocal rather than automatically positive, prevents diagnostic overcall and supports selective, stepwise imaging strategies [38,44].

**Figure 1:**
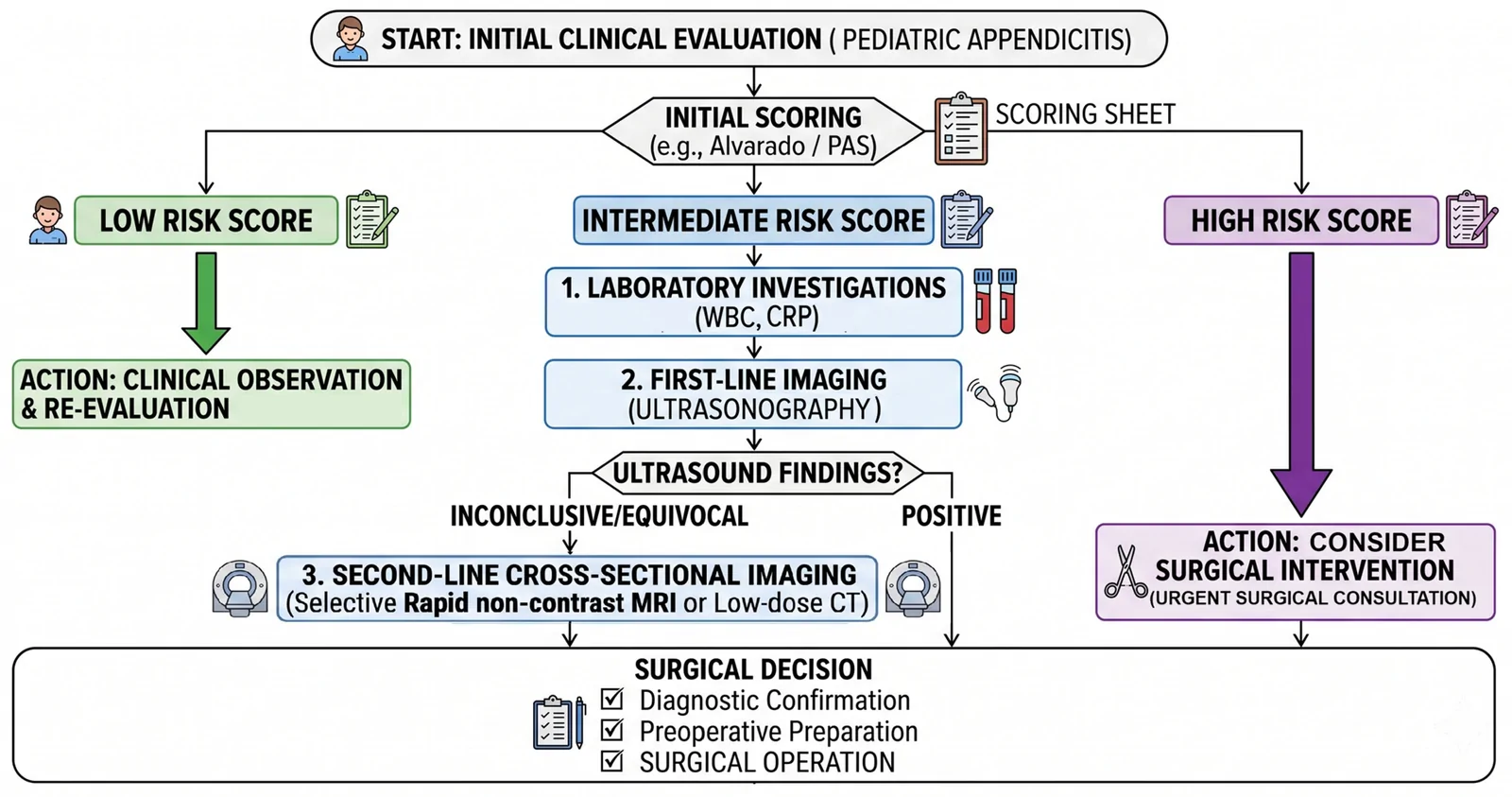
Simplified diagnostic algorithm for the evaluation of acute appendicitis in children.

A simplified diagnostic pathway may be proposed based on the current evidence (Figure 1). Initial assessment should include clinical evaluation and the application of a scoring system such as the Alvarado score or PAS. Patients with low-risk scores may be safely observed with clinical reassessment. In intermediate-risk cases, additional laboratory investigations, including C-reactive protein and white blood cell count, are recommended, followed by ultrasonography as the first-line imaging modality. In cases with inconclusive findings, computed tomography may be considered to clarify the diagnosis. Patients with high-risk scores should be considered for prompt surgical intervention without delay.

A key finding of this review is that the integration of clinical scoring systems, laboratory biomarkers, and imaging modalities contributes to improved diagnostic precision. This multimodal strategy enables effective risk stratification and supports clinical decision-making. From a practical perspective, patients with low-risk scores may be safely observed, while high-risk patients may proceed directly to surgical management. Intermediate-risk patients appear to benefit the most from additional laboratory testing and imaging [24,31].

Importantly, diagnostic performance varies across patient subgroups. Younger children represent the most challenging population due to atypical presentation and rapid progression to complicated disease, predominantly frank perforation and localized or generalized peritonitis [4,9]. Additionally, differences in sex and disease stage may influence the accuracy of diagnostic tools, further supporting the need for individualized approaches. Age-related communication barriers represent a major challenge when applying scoring systems. Clinical scores rely heavily on subjective symptoms (e.g., migration of pain, anorexia, or localized tenderness) that are notoriously difficult to elicit in toddlers and preverbal children. Consequently, clinical prediction scores demonstrate lower accuracy and higher diagnostic uncertainty in this younger age group, emphasizing the reliance on objective laboratory biomarkers and early expert ultrasonography.

Clinical decision rules and risk stratification models represent an important advancement in this field. The clinical decision rule proposed by Kharbanda et al. has been shown to be useful in identifying low-risk patients and may contribute to reducing unnecessary imaging and interventions [43]. These models may support the development of more standardized and efficient diagnostic pathways.

Emerging technologies, including artificial intelligence and machine learning algorithms, offer promising opportunities for improving diagnostic performance. By integrating clinical, laboratory, and imaging data, these tools may enhance predictive performance beyond traditional scoring systems. However, further validation in pediatric populations is required before widespread clinical implementation.

Several limitations of this review should be acknowledged. The heterogeneity of the included studies, variations in study populations, and differences in diagnostic criteria limit the comparability of results. Alongside this, most of the available evidence is derived from observational studies, which may introduce selection bias. The narrative design of this review represents an additional limitation; however, a structured selection approach was applied to ensure the inclusion of relevant and high-quality studies.

In summary, while traditional tools like the Alvarado score provide accessibility for initial triage, optimal diagnostic precision in pediatric acute appendicitis requires a structured, stepwise protocol. Integrating validated scoring systems with targeted inflammatory biomarkers and selective, non-ionizing imaging minimizes clinical ambiguity, optimizes resource utilization, and prevents treatment delays in young children.

## 5. Conclusions

No single clinical score provides sufficient diagnostic accuracy across all pediatric age groups. While the Alvarado score serves as an accessible initial screening tool, its limited specificity precludes standalone use. Pediatric-derived systems like PAS and continuous models such as pARC offer superior risk stratification in children. Incorporating clinical scoring, inflammatory biomarkers, and selective ultrasonography within a stepwise diagnostic algorithm improves accuracy, optimizes imaging utilization, and supports timely surgical decision-making in pediatric appendicitis.

## Figures and Tables

**Table 1:** Reported Diagnostic Performance Ranges of Appendicitis Scoring Systems in Pediatric Validation Cohorts.

Score	Standard Cut-off Threshold	Sensitivity Range (%)	Specificity Range (%)	Primary Clinical Characteristics	References
Alvarado	≥7	72–88	57–76	High sensitivity; limited specificity in children; useful initial screening tool	[10,13,14,20]
PAS	≥7	80–95	60–70	High sensitivity; incorporates pediatric-specific symptoms (cough/hopping pain)	[9,15,19,20]
AIR	≥5	85–92	78–90	Balanced diagnostic profile; incorporates serial CRP; high specificity for complicated disease	[16,21,22,23,24]
RIPASA	≥7.5	88–96	70–88	High sensitivity; includes demographic factors; primary validation in adult/mixed cohorts	[18,25,26]
Tzanakis	≥8	85–96	75–90	Combines clinical signs, WBC count, and ultrasound findings; operator dependent	[1,17]

Note: Data represent reported ranges observed across pediatric-specific validation cohorts evaluated at standard published cut-off thresholds. These values reflect discrete integer scoring systems and are presented to illustrate diagnostic variability rather than a formal statistical meta-analysis. Continuous probability models, such as the Pediatric Appendicitis Risk Calculator (pARC), are excluded from simple range comparisons because they calculate individual risk percentages rather than applying fixed score cut-offs.

**Table 2:** Detailed Parameter Scoring and Diagnostic Thresholds across Clinical Risk Scores.

Predictor/Variable	Measurement Units	Scoring Criteria/Points Assigned	Alvarado (Max 10)	PAS (Max 10)	AIR (Max 12)	RIPASA (Max 17.5)	Tzanakis (Max 15)
Migration of pain to RLQ	Presence (Yes/No)	Yes = 1	1	1	-	0.5	-
Anorexia	Presence (Yes/No)	Yes = 1	1	1	-	1	-
Nausea/Vomiting	Presence (Yes/No)	Yes = 1	1	1	1 (Vomiting)	1	-
RLQ Tenderness	Presence (Yes/No)	Grade/Presence	2	2	1 (Mild), 2 (Mod), 3 (Severe)	1	4
Rebound Tenderness	Presence (Yes/No)	Grade/Presence	1	-	1 (Mild), 2 (Mod), 3 (Severe)	1	3
Muscular Guarding	Presence (Yes/No)	Yes = 1–2	-	1	-	2	-
Cough/Percussion/Hopping Pain	Presence (Yes/No)	Yes = 1–2	-	2	-	-	-
Fever/Temperature	°C	≥37.3 °C (Alvarado)/ ≥38.0 °C (PAS/AIR)/ 37.0–39.9 °C (RIPASA)	1	1	1	1	-
Leukocytosis (WBC)	×10^9^/L	≥10.0 (Alvarado/PAS)/ 10.0–14.9 (AIR/RIPASA)/ ≥15.0 (AIR)	2	1	1 (10.0–14.9), 2 (≥15.0)	1 (10.0–14.9)	2 (≥12.0)
Neutrophilia (PMN)	%	>75% (Alvarado/PAS)/70–84% (AIR)/≥85% (AIR)	1	1	1 (70–84%), 2 (≥85%)	-	-
C-Reactive Protein (CRP)	mg/L	10–49 mg/L (AIR)/ ≥50 mg/L (AIR)	-	-	1 (10–49), 2 (≥50)	-	-
Ultrasound Confirmation	Positive/Negative	Positive for Appendicitis	-	-	-	-	6
Demographics (Age & Sex)	Age (Years), Sex	Age < 13.9 yr (RIPASA)/Male (RIPASA)	-	-	-	1 (<13.9 yr), 1 (Male)	-
Duration of Symptoms	Hours	<48 h (RIPASA)	-	-	-	1 (<48 h)	-
RLQ Guarding/Rovsing Sign	Presence (Yes/No)	Yes = 1–2	-	-	-	2 (Rovsing)	-
Low Risk (Observation/Discharge)	Integer Score	Diagnostic Interpretation Cut-off	<5	≤3	0–4	<7.0	<8
Intermediate Risk (Active Observation/US)	Integer Score	Diagnostic Interpretation Cut-off	5–6	4–7	5–8	7.5–11.5	8–11
High Risk (Surgical Consultation)	Integer Score	Diagnostic Interpretation Cut-off	≥7	≥8	9–12	≥12.0	≥12

Note: Thresholds and predictor definitions represent original derivation parameters and pediatric validation adaptations [12,15,16,17,18]. Abbreviations: RLQ = Right Lower Quadrant; WBC = White Blood Cell count; PMN = Polymorphonuclear neutrophils; CRP = C-reactive protein.

**Table 3:** Clinical Utility and Diagnostic Performance of Biomarkers and Imaging Modalities in Pediatric Appendicitis.

Diagnostic Parameter/Modality	Diagnostic Utility & Clinical Thresholds	Specific Limitations in Pediatrics
White Blood Cell Count	Leukocytosis ($>11.0\times 10^{9}/L$) serves as an early, sensitive screening marker; elevated levels correlate with symptom onset.	Low specificity; frequently elevated in common pediatric viral illnesses and mesenteric adenitis.
C-Reactive Protein	Diagnostic indicator of inflammatory severity (>10 mg/L); levels > 50 mg/L strongly correlate with appendiceal necrosis, perforation, or abscess formation.	Delayed kinetics (peaks 24–48 h post-onset); low sensitivity in early (<12 h) presentation.
Neutrophil-to-Lymphocyte Ratio	Calculated ratio (typically >3.5–4.5) enhancing predictive accuracy when combined with clinical risk scores (AIR, MAS).	Thresholds vary across pediatric age brackets; requires absolute cell count verification.
Ultrasonography	First-line imaging tool; evaluates appendiceal diameter, wall compressibility, mucosal hyperemia, and peri-appendiceal fat change.	Highly operator-dependent; limited by patient body habitus, bowel gas, and non-visualization of the appendix.
Computed Tomography	Diagnostic sensitivity and specificity > 95%; second-line modality reserved for equivocal clinical/ultrasound findings.	Exposure to ionizing radiation; requires judicious, protocolized application to avoid unnecessary radiation risk.

Note: Data synthesized from pediatric evidence evaluating laboratory kinetics and imaging accuracy [11,27,28,29,30,31,37,38,43].

## Data Availability

No new data were created or analyzed in this study. Data sharing is not applicable to this article.

## Abbreviations

The following abbreviations are used in this manuscript:

AIR	Appendicitis Inflammatory Response
CRP	C-Reactive Protein (expressed in mg/L)
CT	Computed Tomography
MAS	Modified Alvarado Score
MRI	Magnetic Resonance Imaging
NLR	Neutrophil-to-Lymphocyte Ratio
pARC	Pediatric Appendicitis Risk Calculator
PAS	Pediatric Appendicitis Score
PMN	Polymorphonuclear Neutrophils
RIPASA	Raja-Isteri-Pengiran-Anak-Saleha
RLQ	Right Lower Quadrant
WBC	White Blood Cell Count (expressed in ×10^9^/L)
